# Research on the safety evaluation of children's activity space in urban residential areas

**DOI:** 10.3389/fpubh.2026.1832723

**Published:** 2026-06-17

**Authors:** Yang Zhou, Meng Wang, Caiyun Qian, Xinye Shen

**Affiliations:** 1 School of Architecture, Soochow University, Suzhou, China; 2 School of Architecture, Nanjing Tech University, Nanjing, China

**Keywords:** built environment, children's activity spaces, perceived safety, social environment, structural equation modeling, urban residential areas

## Abstract

**Introduction:**

Residential communities constitute important settings for children's daily outdoor activities, and the perceived safety of outdoor activity spaces among parents and children plays a critical role in shaping children's outdoor activity behaviors. However, rapid urbanization, increasing traffic volumes, and large-scale urban construction have reduced opportunities for safe outdoor activities, contributing to a decline in children's independent mobility and potentially affecting their healthy development. This study aims to identify the key factors influencing users' safety perceptions of children's outdoor activity spaces in residential communities.

**Methods:**

Based on a comprehensive literature review and user satisfaction evaluations of the safety conditions of children's outdoor activity spaces, this study developed a safety perception evaluation framework comprising five dimensions: site safety, management safety, neighborhood safety, facility safety, and environmental safety. Exploratory factor analysis (EFA), first-order confirmatory factor analysis (CFA), and second-order CFA were employed to examine the underlying factor structure and relationships among safety perception dimensions.

**Results:**

The results indicated that site safety and facility safety carried the greatest weights among the five dimensions. Physical characteristics, including spatial layout, site boundaries, activity organization, and facility provision, were identified as the core factors influencing users' safety perceptions. In addition, strict and well-organized community management, together with a harmonious and supportive neighborhood environment, significantly enhanced residents' trust in environmental safety and increased their willingness to support children's outdoor activities. The second-order factor model further confirmed the multidimensional structure of safety perception in residential outdoor activity spaces for children.

**Discussion:**

The findings provide a comprehensive framework for evaluating the safety of children's outdoor activity spaces, reflecting the psychological perceptions and subjective feelings of safety among both parents and children. The proposed evaluation system can assist planners, designers, and community managers in identifying critical safety deficiencies, informing targeted interventions, and supporting future research on the safety and health-promoting potential of residential outdoor environments for children.

## 1 Introduction

With the progression of urbanization, China's urban development has shifted from emphasizing “quantity” to “quality,” and from focusing on “objects” to focusing on “people.” Fully addressing the daily spatial needs of different resident groups has become an inevitable requirement in this new phase of urban development. Children are the most frequent users of outdoor spaces in residential areas, and a dialectical relationship of interdependence and mutual influence exists between them and their residential environments: residential space is not only the physical setting for children's daily lives but also an educational mechanism within the socio-spatial field, directly impacting children's physical and mental health (1), behavior guidance (2, 3), and personality development (4). Over the past two decades, rapid motorization has significantly encroached upon traditional residential street spaces, high-density land development has promoted the vertical expansion of residential spaces, and the proliferation of gated commodity housing estates has contributed to the enclosed management of neighborhood spaces. Consequently, child-friendly urban spaces have exhibited characteristics of fragmentation, homogenization, enclosure, and even disappearance, highlighting the contradiction between children's daily behavioral needs and the configuration models of public space. Furthermore, increased academic pressure due to educational demands and the pervasive influence of digital media have led to more indoor, electronic, and sedentary lifestyles among children, significantly reducing their independent outdoor activities and physical activity levels (93).

Prolonged lack of outdoor activity can not only lead to various physical health issues in children, such as decreased physical fitness, impaired eyesight, and obesity (5, 6), but can also cause negative psychological emotions like loneliness and depression (7). As one of the countries with the largest child population globally, creating urban environments in China that meet children's needs and ensure the safety of their activities has become a key focus in contemporary urban design. To safeguard children's rights to “freely play, relax, and engage in recreation,” the state has issued a series of documents, including the China Children's Development Outline (2011–2020), the Specifications for the Construction of Child-Friendly Communities, and the Guiding Opinions on Promoting the Construction of Child-Friendly Cities. However, outdoor spaces for children within existing residential areas still suffer from issues such as inadequate spatial dimensions, safety hazards in spatial layout, and insufficient community governance and management. Some scholars, through surveys, suggest that old urban areas generally lack sufficient and equitably distributed outdoor spaces for children; while new districts and newly built residential complexes often provide dedicated areas, these tend to rely on simplistic play equipment, are disconnected from nature, and exhibit characteristics of low standards and deviation (8, 9). Beyond the objective built-environment issues mentioned above, such as the insufficient quantity and poor quality of community open spaces, residential safety hazards, and the perception of safety are also major factors contributing to the decline in children's outdoor activities. Existing research indicates that parents' perception of neighborhood safety influences their children's activity levels (10–12), and that safe play environments are conducive to children's play activities (13), enhancing their cognitive abilities, emotional wellbeing, and social skills (14). Therefore, improving the safety and perceived safety of children's outdoor activity spaces in residential areas is crucial for promoting children's activity and healthy development. Research on the perception of safety in children's outdoor activity spaces within residential areas serves as a critical pathway to understanding the relationship between children's daily activities and their environment. It holds significant importance for both promoting children's outdoor engagement and leveraging the essential “educational” function of urban community spaces. Drawing on a comprehensive literature review, this study identifies built environmental and social environmental factors that influence the perceived safety of outdoor activity spaces. An investigation was conducted into the characteristics of children's outdoor activities in residential areas of Nanjing's main urban districts, along with an assessment of parental and children's satisfaction regarding the current safety conditions of these spaces. Utilizing SPSS 22.0 and AMOS 21.0 software, a structural equation model was employed to develop a subjective evaluation system for the safety of children's outdoor activity spaces in residential settings. This research examines the relationship between environmental elements of these spaces and users' subjective perceived safety. Based on the findings, optimization strategies are proposed to enhance the safety of existing residential children's activity spaces, thereby fostering children's routine outdoor engagement and supporting the development of “child-friendly communities.” This study aims to provide empirical evidence for improving the level of perceived safety in residential community spaces, while offering decision-making references and theoretical support for enhancing children's outdoor activity levels and promoting public health.

## 2 Literature review

### 2.1 Theoretical background of safe community and CPTED

The concept of the “Safe Community” was first proposed in the late 1980s. Its core principle is to create a physical and social environment that maximizes the prevention of various injuries (including both unintentional and intentional injuries) and ensures the safety and health of people of all age groups in all living scenarios through the joint participation and cooperation of community residents (15). Research on residents' perception of community safety has shown that factors such as the degree of resident participation (16), neighborhood social relations (17, 18), and community management and governance (19, 20) all influence residents' perceived safety, which in turn affects their activities within the community. Regarding children's perceived safety and parents' permission for independent mobility, some studies have found that parental concerns about their children's independent travel are formed by a complex interaction of personal, social, and environmental factors. The presence of companions, favorable environmental conditions, and the emphasis on positive outcomes may, to some extent, alleviate parental worries (21, 22).Relevant cross-sectional data indicate that fear of strangers, traffic accidents, and unintentional injuries are often key factors determining whether parents allow their children to move around in community spaces (23, 24), while green spaces, supervision facilities, and recreational amenities can enhance parents' perception of community safety (20, 25).

CPTED theory (Crime Prevention Through Environmental Design) is a crime prevention theory based on the interaction between environmental design and human behavior, aimed at reducing opportunities for crime through spatial planning, architectural design, and other means. This theory initially derived from the concept of “eyes on the street” proposed by Jacobs in The Death and Life of Great American Cities (26), suggesting that increased pedestrian flow brings more natural surveillance to the streets, enhances mutual monitoring among people, and improves area safety (27). In the 1970s, Jeffery formally introduced the CPTED concept in Crime Prevention Through Environmental Design (28). Subsequently, academia summarized six key elements of CPTED theory from the related theories of scholars like Jacobs, Newman (29), Territoriality, Natural Surveillance, Access Control, Target Hardening, Image/Maintenance, and Activity Support, which have gained widespread recognition. Later research based on CPTED and Defensible Space theory has also extensively considered social environmental factors (30). Numerous case studies have demonstrated the effectiveness of CPTED theory in preventing community crime and enhancing community safety (31–33), providing significant guidance for the safety design of community environments. Existing research on CPTED theory in China has primarily focused on the relationship between individual factors, management factors, and residential safety (34, 35), while studies on the safety of the built environment in communities are relatively scarce (27).

### 2.2 Relationships among residential environment, perceived safety, and outdoor activity levels

**Figure 1:**
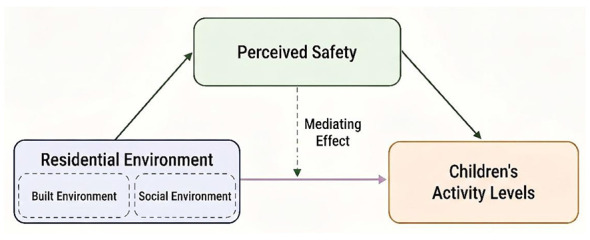
Relationships among residential environment, perceived safety, and children's activity levels in previous studies.

Existing studies have systematically examined the associations among residential environment, perceived safety and children's outdoor activity levels from multiple dimensions (Figure 1). The main research themes include the direct effects of residential environment on children's outdoor activity levels, the interaction mechanisms and mediating relationships among the three variables, as well as the pathways through which residential environment influences perceived safety.

Regarding the direct relationship between residential environment and children's outdoor activity levels, a substantial body of empirical research has demonstrated that the residential built environment is a key determinant of children's outdoor activities, although its underlying mechanisms exhibit considerable complexity and contextual dependency (36, 70). Studies employing correlation analyses and regression models have found that variables such as natural environmental attributes, higher walkability, close neighborhood ties, and site safety are all significantly and positively associated with children's activity levels (37–39, 94). Specifically, the relevant indicator systems mainly encompass physical environmental factors (40), characteristics of activity spaces (41, 42), provision of activity facilities (43), landscape environment (44), and social environmental factors such as neighborhood relationships (45). Furthermore, the effects of environmental factors vary significantly across different developmental stages and geographical contexts, with particularly evident spatial heterogeneity between developed and developing countries (46).

Some studies have explored the interrelationships among the built environment, perceived safety, and children's activity levels, with findings suggesting that perceived safety plays a critical mediating or moderating role between residential environment and children's activities. Its influence pathway is typically characterized by a chain mechanism of “environmental characteristics–perceived safety–behavioral response.” Further studies have indicated that this process may also be subject to multiple mediating or moderating effects from factors such as social environment and peer interaction, thereby forming a complex multi-path influence structure (47–49). Lin et al. (50) found that parents' perceptions of neighborhood cohesion and social relationships, as well as their perceptions of traffic risks, significantly influence their evaluations of overall residential safety, which in turn affects children's levels of independent activity. Tappe et al. (47) likewise reported that different dimensions of perceived safety, such as traffic safety and crime safety, are closely associated with children's activity levels. D'Haese et al. (22) suggested that lower parental concerns regarding traffic safety and crime safety are conducive to children's physical activity in residential streets and public spaces. Zhang et al. (51) further proposed that perceived built-environment variables exert a moderate mediating effect on the relationship between the built environment and physical activity.

With regard to the relationship between residential environment and perceived safety, a close intrinsic connection exists between the two. Existing studies generally suggest that perceived safety is a key prerequisite influencing whether children are able to engage in independent outdoor activities and achieve higher activity levels (52–56) pointed out that young children's frequency of activities in natural environments is significantly influenced by parents' perceptions of safety and their permission for outdoor behavior. Based on an empirical study of children aged 11–15 years, Janssen (57) found that children's subjective perceptions of neighborhood safety were directly and positively associated with their participation in outdoor activities. Factors influencing perceived safety mainly include potential crime risks, heavy traffic, environmental design quality, and surrounding residential spatial characteristics (12, 58). For example, Gao et al. (59) found that residential floor area ratio has a positive effect on children's perceived safety, with a stronger influence than the proportion of green space. Moreover, compared with the physical environment, some studies have suggested that social environmental factors—such as policy and institutional settings, sociocultural atmosphere, neighborhood interaction, and peer companionship—may exert a more significant influence on perceived safety and children's independent activities (59, 60, 70).

In recent years, with the development of big-data acquisition techniques and machine learning methods, studies based on street-view imagery and computer vision technologies have gradually emerged. Some studies have quantitatively assessed place-based perceived safety among different population groups, such as women, older adults, and university students, through semantic segmentation and feature extraction, identifying environmental factors closely associated with perceived safety, including landscape view index, level of motorization, and openness of pedestrian spaces (61–64). These approaches complexity, green provide a new technical pathway for quantitative modeling from objective environmental characteristics to subjective perceptions.

### 2.3 Perceived safety scale

In the field of subjective measurement of spatial safety perception, several influential scales have gradually been developed, mainly including the Neighborhood Safety Scale, the Neighborhood Environment Walkability Scale (NEWS), the CPTED Perception Scale, and the Public Open Space Safety (POS Safety) Scale. These scales differ in both theoretical foundations and measurement dimensions. Among them, the Neighborhood Safety Scale has been widely applied in public health and environmental behavior research. Its core dimensions generally include fear of crime, safety at night, social trust, and safety for children, emphasizing individuals' comprehensive perceptions of the residential social environment and potential risks (65). The NEWS scale primarily focuses on the supportive role of the built environment in walking and physical activity. Structurally, it emphasizes a dual-dimensional framework of “traffic safety and crime safety,” with specific contents involving destination accessibility, street connectivity, safety of pedestrian facilities, perceived crime risk, and environmental aesthetics (66, 67). It is currently one of the most widely used environmental perception measurement tools internationally, although its relatively large number of items may create certain difficulties in survey implementation. The POS Safety Scale is theoretically closely associated with CPTED. Its typical dimensions include Natural Surveillance, Lighting and Visibility, Access and Escape, Maintenance/Order, and Activity and Social Presence, placing greater emphasis on safety experiences within public open space contexts (68).

The above scales differ in content emphasis and analytical perspectives. The CPTED Scale and POS Safety Scale focus more on the relationship between criminal behavior and environmental design, and therefore include a greater number of spatial perception indicators related to fear of crime, natural surveillance, and nighttime lighting. By contrast, the NEWS Scale has been more widely adopted internationally, but its perspective is largely centered on walking environments; accordingly, its items involve destination accessibility, facility provision within walking distance, and street network connectivity. Overall, although substantial overlap exists among these scales in terms of content composition, no universally standardized questionnaire system applicable across contexts has yet been established due to differences in research populations, spatial types, and contextual requirements. Consequently, existing studies have generally conducted contextual revisions and reconstructions based on the core dimensions of classical scales to accommodate the differentiated measurement needs of specific population groups (e.g., women, adolescents, and older adults) and specific spatial settings (e.g., streets, parks, and campuses) (69).

In summary, empirical evidence regarding how the built environment and neighborhood social environment jointly influence individuals' perceived safety in urban residential activity spaces remains relatively limited. In particular, systematic understanding of the mechanisms through which environmental factors shape perceived safety among children and their parents still requires further development. In existing studies, some literature has simplified perceived safety as a defensive perception of crime risk, overlooking its broader and multidimensional conceptual structure. In the context of children's outdoor activities, perceived safety not only involves fear of crime, but also encompasses comprehensive judgments of various potential risks embedded within both physical and social environments. These include the safety of activity spaces and facilities themselves, the safety conditions of surrounding traffic environments, and socially induced risks arising from neighborhood interactions, such as bullying behaviors of older children toward younger children. Most existing scales have been developed based on specific research perspectives and target populations, resulting in different emphases in the selection of perceived safety dimensions, such as environmental support for walkability or crime-prevention-oriented spatial attributes. Although these scales provide important references for the present study, there remains a lack of universally applicable instruments specifically suited to the context of “children's outdoor activity spaces in urban residential communities.” At the same time, some classical scales contain relatively lengthy and complex items, which objectively increase survey burden and constrain their practical applicability in field investigations. Moreover, perceived safety exhibits substantial variation across different geographical and institutional contexts. Compared with the open residential environments commonly found in Western countries, urban residential communities in China are generally characterized by enclosed or semi-enclosed spatial management patterns and relatively high levels of public security. This contextual background determines that both the influencing factors and the pathways shaping perceived safety possess strong contextual specificity. Based on existing theoretical frameworks and measurement scales, this study therefore constructs a subjective evaluation system for perceived safety in children's outdoor activity spaces within the context of Chinese urban residential communities, or communities with similar spatial characteristics. The study aims to systematically identify the key environmental factors influencing the perceived safety of children and their parents, as well as the interrelated mechanisms through which these factors operate.

## 3 Methods

Based on the theoretical assumption that perceived safety is a multidimensional psychological perception rather than a single observable attribute, this study proposes a second-order conceptual framework for the evaluation of children's outdoor activity space safety. This study conceptualizes perceived safety in children's outdoor activity spaces as a hierarchical multidimensional construct. The proposed conceptual framework consists of three levels: observed variables, first-order latent constructs, and a higher-order perceived safety construct. Multiple observed indicators derived from questionnaire items are assumed to reflect several underlying first-order dimensions, which collectively constitute the second-order latent construct of perceived safety. Exploratory factor analysis (EFA) was first employed to identify the latent dimensional structure, followed by confirmatory factor analysis (CFA) to validate the hierarchical conceptual model and establish the perceived safety evaluation system. Specifically, this study employed a combination of literature review, theoretical analysis, and field surveys/interviews to identify observable variables influencing users' perceived safety in children's outdoor activity spaces within residential communities. A questionnaire was constructed using a Likert scale to gather evaluations from parents and children regarding their satisfaction with the current safety conditions of these spaces. Subsequently, Exploratory Factor Analysis (EFA) was conducted using SPSS software to screen variables affecting users' psychological perception and safety evaluations, forming new factor groups (latent variables) through dimensionality reduction. Then, employing AMOS software, a Confirmatory Factor Analysis (CFA) model was established to define the relationships between observable and latent variables, thereby constructing a user-centered safety evaluation system for children's outdoor activity spaces in residential areas. Finally, based on the impact relationships within the evaluation model, the weight of each indicator was calculated, and recommendations for enhancing the safety of these spaces were proposed.

### 3.1 Questionnaire design and indicator selection

The questionnaire comprised three sections: basic resident information, characteristics of children's activities, and evaluation of perceived safety in residential children's outdoor activity spaces. The residents' basic information included children's age and gender, as well as parents' age and gender. Children's activity characteristics included activity frequency, activity duration, and safety supervision methods.

Based on the aforementioned theoretical analysis (safe community theory and CPTED theory), existing literature review, and the content of the Perceived Safety Scale, this study divides perceived safety in children's outdoor activity spaces into built environment safety and neighborhood social environment safety. Built environment safety primarily refers to the sense of security derived from the actual physical environment—including sites, facilities, and surroundings—as experienced by residents during use. This encompasses considerations such as the comfort, cleanliness, aesthetic quality of the spaces, and the intrinsic safety of the facilities. Neighborhood social environment safety mainly includes two aspects: firstly, perception of interpersonal friendliness within the living circle, based on neighborly relations, i.e., concerns about neighborhood safety, including neighborhood harmony and unease around strangers (70); secondly, perception of social safety factors within the living environment, based on residential property management, i.e., concerns about environmental hazards, including traffic control and surveillance (42, 71).

**Figure 2:**
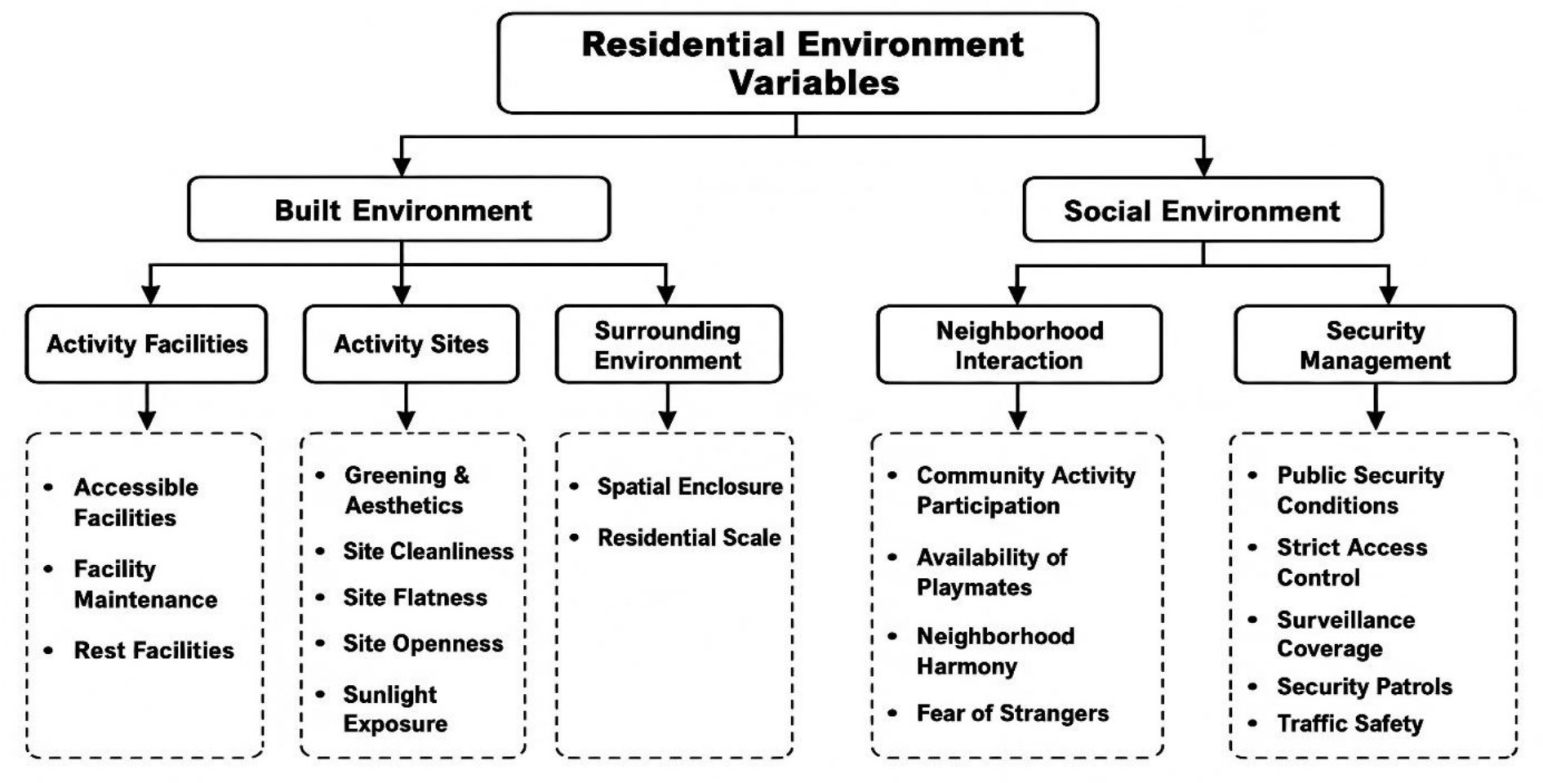
Conceptual structure of perceived safety variables.

Considering that residential communities in Chinese cities are commonly composed of multi-story or high-rise housing complexes, are spatially enclosed by walls or similar boundaries, and are mostly under gated management, this study screened and integrated safety perception factors identified in previous literature when designing the questionnaire. Built environment safety-related variables were categorized into three dimensions: activity facilities, activity sites, and surrounding environment, corresponding, respectively to accessible facilities, facility maintenance, and rest facilities; greening & aesthetics, site cleanliness, site flatness, site openness, and sunlight exposure; as well as spatial enclosure and residential scale, resulting in a total of 10 variables. In addition, social environment safety-related variables were divided into two dimensions: neighborhood interaction and security management, corresponding, respectively to community activity participation, availability of playmates, neighborhood harmony, and fear of strangers; as well as public security conditions, strict access control, surveillance coverage, security patrols, and traffic safety, resulting in a total of 9 variables (Figure 2). A Likert scale (1 = Very Dissatisfied to 5 = Very Satisfied) was used to measure each variable. The sources and explanations of these variables are listed in Table 1.

**Table 1:** Description and sources of observed variables in the questionnaire.

Category	No.	Observed variable	Variable description	Source
Activity facilities	1	Accessibility facilities	Completeness of (barrier-free facilities) in children's outdoor activity spaces, including continuous ramps (e.g., allowing stroller access).	(66, 72)
	2	Facility maintenance	Regular maintenance of activity equipment and facilities by dedicated personnel within the residential area; timeliness of repairing damaged equipment.	(14, 94)
	3	Rest facilities	Adequacy of rest facilities (e.g., seating) within the residential children's outdoor activity spaces to meet daily needs.	(20, 25, 94)
Activity sites	4	Greening & aesthetics	Aesthetic quality of landscaping within the children's outdoor activity spaces and maintenance of flora by sanitation services.	(25, 38, 61, 63)
	5	Site cleanliness	Cleanliness of the environment within the children's outdoor activity spaces, including absence of (garbage) or leaf piles, and adequacy of trash bins.	(68, 73)
	6	Site flatness	Absence of significant level differences within the activity space, ensuring strong site continuity.	(66, 94)
	7	Site openness	Spaciousness and openness of the children's outdoor activity space, providing good visual permeability and absence of obstructions.	(63, 94)
	8	Sunlight exposure	Adequacy of sunlight within the children's outdoor activity space, ensuring a suitable environment (particularly during winter).	(63, 74)
Surrounding environment	9	Spatial enclosure	Sense of spatial enclosure within the children's outdoor activity space; comfort level of boundary treatments (e.g., fences, greenery, walls).	(68, 75)
	10	Residential scale	Number of buildings and population size within the residential community.	(20, 51, 59)
Neighborhood Interaction	11	Community activity participation	Frequency of residents' use of outdoor activity spaces and their participation level in neighborhood activities within the residential community.	(17, 59, 60)
	12	Availability of playmates	Presence of (similar-aged) playmates for children in the residential outdoor activity spaces.	(10, 60, 70)
	13	Neighborhood harmony	Existence of acquainted neighbors and a harmonious relationship among them.	(13, 25, 40, 65)
	14	Fear of stranger	Level of concern among parents and children regarding unfamiliar individuals within the residential area.	(12, 24)
Security Management	15	Public security conditions	Status of security management within and around the residential area (e.g., occurrence of theft, robbery, abduction, and other criminal activities).	(12, 19, 23, 68)
	16	Strict access control	Completeness of the residential access control system and strictness of security guards, preventing non-residents from entering freely.	(28, 29, 34, 76)
	17	Surveillance coverage	Comprehensiveness of surveillance camera coverage around children's activity spaces.	(68, 77, 78)
	18	Security patrols	Regularity of security patrols within the residential area.	(29, 40)
	19	Traffic safety	Traffic safety conditions on routes to activity spaces for children; vehicle parking situation around the sites.	(12, 13, 24)

### 3.2 Survey overview

The survey targeted children and their guardians in residential communities. Given that most children engaging in outdoor activities are of preschool and elementary school age with limited reading comprehension abilities, guardians were instructed to complete the questionnaire based on their observations and their children's feedback during outdoor activities. The questionnaire survey adopted a combination of random sampling and convenience sampling methods. With the assistance of the online survey platform Wenjuanxing, questionnaires were distributed through both online and field-based approaches to kindergarten and primary school parent groups within residential communities in the Longjiang area of Nanjing, as well as to children and parents engaging in activities within residential community spaces. The questionnaire survey adopted a combination of random sampling and convenience sampling methods. With the assistance of the online survey platform Wenjuanxing, questionnaires were distributed through both online and field-based approaches to kindergarten and primary school parent groups within residential communities in the Longjiang area of Nanjing, as well as to children and parents engaging in activities within residential community spaces. This study planned to employ Structural Equation Modeling (SEM) for data analysis. According to methodological requirements, the model contained 19 observed variables. Based on the estimated number of free parameters in the model structure and the commonly recommended sample-to-parameter ratio of 10:1, while also satisfying the minimum sample size requirement of at least 200 cases (79–82), the minimum required effective sample size was estimated to be 400 questionnaires, with an ideal range of 500–800 questionnaires considering model complexity and estimation stability.

A total of 515 electronic questionnaires were collected, including 421 online questionnaires and 94 field-based convenience sampling questionnaires, achieving the predetermined target sample size. Data screening followed these criteria: completion time under 120 s (five parents were invited to participate in a pilot survey, with completion times ranging from 200 to 250 s and an average of 230 s; based on the commonly adopted empirical threshold of 40%−50% in social science research, questionnaires completed in less than 110 s were considered invalid in this study), respondents not matching the target demographic (e.g., respondents who were not the child's primary daily guardian and therefore could not ensure response accuracy), and patterned responses (e.g., straight-line answers). After screening, 432 valid questionnaires were retained, yielding an effective response rate of 83.88%.

### 3.3 Evaluation model development

Through a review of the relevant literature, existing quantitative studies on subjective perceptions of children's outdoor activity spaces have mainly employed methods such as correlation analysis, linear regression, logistic regression, analytic hierarchy process (AHP), and Structural Equation Modeling (SEM). Given that the safety evaluation of children's outdoor activity spaces involves a large number of observed variables and potential interrelationships among variables, the evaluation framework includes not only directly measurable observed variables but also latent variables that cannot be directly measured. Moreover, the relationships among variables exhibit complex characteristics involving multiple pathways and hierarchical levels. In this context, and considering that this study aims to explore the influencing factors and underlying mechanisms of perceived safety in children's outdoor activity spaces, SEM demonstrates stronger applicability. Compared with traditional regression methods, SEM can effectively control for measurement errors and is particularly suitable for latent variables, path analysis among variables, and the interpretation of underlying mechanisms (79, 83). Structural Equation Modeling integrates factor analysis and path analysis and consists of two components: the measurement model and the structural model. The former is used to characterize the relationships between latent variables and observed indicators, whereas the latter is used to represent the structural relationships among latent variables.

This study utilizes SEM to develop a safety evaluation model for children's outdoor activity spaces in urban residential areas. Based on an analysis of the components constituting the safety evaluation system, the model is constructed across five dimensions: site safety, management safety, neighborhood safety, facility safety, and environmental safety. These five dimensions serve as latent variables, with the 19 evaluation items under these dimensions acting as observable variables. The analysis examines the influence pathways among variables, as well as the influence relationships and weights between the latent and observable variables, ultimately yielding the evaluation model.

First, to investigate parents' and children's fundamental perceived safety in these outdoor spaces and the psychological structure underlying their evaluations, this study employs Exploratory Factor Analysis (EFA). EFA is used to condense the multiple observable variables into a smaller set of factor groups. The factor loadings for each group and the cumulative contribution rate of the factor structure are calculated. EFA is a statistical method designed to extract common factors from a set of variables. Its primary objective is to determine the number of factors influencing the observable variables and the degree of correlation between each factor and its corresponding variables. By analyzing the interrelationships among variables, those with strong correlations are grouped together, thereby reducing the number of variables and creating new factors that capture the essential information contained in the original set (84).

Based on the new factor groups obtained from the exploratory factor analysis, a preliminary safety evaluation model for children's outdoor activity spaces in urban residential areas was constructed. The maximum likelihood method was employed to conduct first-order confirmatory factor analysis (CFA). Confirmatory factor analysis can assess the model's rationality and obtain the influence relationships among variables. Through model fit testing and model modification, the final optimal model was obtained.

Finally, the weights of each indicator were calculated to measure the importance of first-order observed variables and second-order latent variables on the safety evaluation of children's outdoor activity spaces in residential areas, determining the impact of each variable on the overall evaluation model. Based on the analysis of the weight values of each indicator, strategic suggestions for improving the safety of children's activity spaces were discussed.

## 4 Results

Prior to conducting the formal analysis, the reliability and validity of the valid questionnaires were tested. Reliability refers to the stability and consistency of measurement results, while validity indicates the effectiveness of the measurement questionnaire—specifically, the extent to which the questionnaire accurately and appropriately measures the target variables (85). The results showed that the Cronbach's α coefficient for reliability was 0.821 (>0.7), indicating high internal consistency. The KMO value for validity was 0.899 (>0.6), and Bartlett's test of sphericity yielded a significance level of *p* = 0.000 (< 0.01), confirming the suitability of the data for factor analysis. These results demonstrate that the questionnaire possesses good reliability and validity.

### 4.1 Basic information and activity characteristics of survey respondents

The demographic characteristics of the respondents are summarized in Table 2. Female children accounted for 54.40% of the sample, while male children comprised 45.60%, indicating a relatively balanced gender distribution. In terms of age distribution, children aged 3–5 years represented 54.63% of the respondents, those aged 6–12 years accounted for 30.55%, and children over 12 years old constituted 10.19%. Overall, the sample was mainly concentrated in the 3–12 age groups. Based on field survey findings, preschool children have not yet fully developed independent mobility, and their outdoor activities are typically accompanied by parents. In contrast, children aged 12 and above tend to participate less frequently in outdoor activities within residential communities, due to factors such as increased academic pressure, shifts in activity preferences, and reduced discretionary time outside school hours. This age distribution pattern is generally consistent with the actual conditions of children's outdoor activities in residential communities observed in field investigations, indicating that the survey sample has good empirical representativeness.

**Table 2:** Descriptive statistical analysis of the basic characteristics of respondents (Sample size: 432).

Category	Variable	Frequency	Percentage (%)	Category	Variable	Frequency	Percentage (%)
Parental age	31–35	179	41.43	Activity frequency	1–2 times	176	40.74
	36–40	112	25.93		3–4 times	98	22.69
	Above 45	59	13.66		Daily	67	15.51
	41–45	49	11.34		Almost never	57	13.19
	25–30	33	7.64		5–6 times	34	7.87
Parental gender	Female	365	84.49	Activity duration	30 min−1 h	200	46.30
	Male	67	15.51		15–30 min	112	25.93
Child's age	3–5	236	54.63		1–2 h	63	14.58
	6–8	104	24.07		15 min	46	10.65
	Above 12	44	10.19		Above 2 h	11	2.54
	9–12	28	6.48	Safety supervision methods	Full accompaniment	331	76.62
	Under 3 years old	20	4.63		Verbal reminders	76	17.59
Child's gender	Female	235	54.40		Carrying a phone or GPS bracelet	21	4.86
	Male	197	45.60		Assistance from acquaintances	4	0.93

The overall reliability of the questionnaire, measured by Cronbach's α coefficient, was 0.821 (>0.7). The validity, assessed by KMO (Kaiser–Meyer–Olkin) measure, was 0.899 (>0.6), with a significance level of p = 0.000 (< 0.01).

The survey results indicate that the majority of children (40.74% of respondents) engage in outdoor activities 1–2 times per week. Some parents reported that due to heavy academic workloads and participation in extracurricular classes, their children only have time for outdoor activities on weekends. Notably, more than half of the surveyed children (53.94%) engage in outdoor activities no more than twice per week ( ≤ 2 times), which falls significantly below recommended levels of physical activity for children. Whether this phenomenon is related to the design of existing outdoor activity spaces in residential areas requires further investigation. In terms of activity duration, 46.30% of children spend 30 min to 1 h per session outdoors, while 82.87% spend less than 1 h per session. These data reveal a severe insufficiency in children's daily outdoor exposure time. In addition to factors such as academic scheduling, this phenomenon may also be associated with the environmental quality of outdoor activity spaces in residential communities and the level of perceived safety. When activity spaces are affected by issues such as inadequate site maintenance, traffic disturbances, weak security management, or heightened parental safety concerns, children's frequency of outdoor activities, and duration of stay may be further constrained. The underlying relationships among these factors warrant further investigation in future research.

Regarding parental supervision during outdoor activities, the data show that the majority of children are accompanied by parents, while only 22.45% play independently with peers without adult supervision. This suggests that children's social interactions during outdoor activities remain largely confined to family circles, with limited opportunities for peer socialization. The data indicate parental concerns about the safety of children's outdoor activities in residential communities, underscoring the constraining role of perceived safety in shaping children's outdoor behaviors.

### 4.2 Descriptive statistics of observed variables

At the level of activity facilities, respondents reported the highest mean satisfaction with rest facilities (3.54 ± 1.22), while accessibility facilities received the lowest mean score (3.08 ± 1.23), indicating that there remains considerable room for improvement in the provision of accessible facilities in residential communities. At the level of activity sites, “sunlight exposure” received the highest satisfaction score (3.78 ± 1.04), suggesting that the layout of children's activity areas has adequately considered environmental conditions such as sunlight exposure. In contrast, “site flatness” received the lowest score (3.34 ± 1.23). Field observations indicate that inadequate routine maintenance in some activity spaces—such as surface depressions, damaged paving tiles, and aging rubber flooring—are the primary factors contributing to lower satisfaction with site flatness. At the level of surrounding environment, satisfaction with “residential scale” (3.46 ± 0.96) and “spatial enclosure” (3.30 ± 1.10) remained at a moderate level overall. However, the relatively large standard deviation for spatial enclosure suggests substantial inter-individual variation in perceived enclosure experience. At the level of neighborhood interaction, the mean satisfaction score was 3.55, the highest among the five dimensions. Among the indicators, “neighborhood harmony” scored the highest (3.90 ± 1.00), while “fear of strangers” received the lowest mean score (2.78 ± 0.76) and the smallest standard deviation, indicating generally positive evaluations of neighborhood relations across the sample. Familiar neighborhood environments and frequent peer interactions may have reduced both children's and parents' concerns about strangers, resulting in a relatively consistent evaluation pattern. By contrast, satisfaction with security management was the lowest among all dimensions, with a mean score of only 3.05. Specifically, items such as “strict access control” (2.56 ± 1.38), “surveillance coverage” (2.38 ± 1.25), and “security patrols” (2.57 ± 1.35) all received relatively low scores, with standard deviations exceeding 1, indicating substantial heterogeneity in respondents' perceptions of residential security management conditions (Table 3).

**Table 3:** Descriptive statistics of variables.

Classification	No.	Classification of observed variables	Response option	Mean (SD)
Activity facilities mean: 3.29	1	Accessibility facilities	1~5; 1 = Very dissatisfied; 5 = Very satisfied.	3.08 (1.23)
	2	Facility maintenance		3.25(1.25)
	3	Rest facilities		3.54(1.22)
Activity sites mean: 3.48	4	Greening &aesthetics	1~5; 1 = Very dissatisfied; 5 = Very satisfied.	3.46(1.16)
	5	Site cleanliness		3.38(1.19)
	6	Site flatness		3.34(1.23)
	7	Site openness		3.45(1.21)
	8	Sunlight exposure		3.78(1.04)
Surrounding environment mean: 3.38	9	Spatial enclosure	1~5; 1 = Very dissatisfied; 5 = Very satisfied.	3.30(1.10)
	10	Residential scale		3.46(0.96)
Neighborhood interaction mean: 3.55	11	Community activity participation	1~5; 1 = Very dissatisfied; 5 = Very satisfied.	3.69(0.99)
	12	Availability of playmates		3.82(1.00)
	13	Neighborhood harmony		3.90(1.00)
	14	Fear of stranger		2.78(0.76)
Security management mean: 3.05	15	Public security conditions	1~5; 1 = Very dissatisfied; 5 = Very satisfied.	3.95(0.84)
	16	Strict access control		2.56(1.38)
	17	Surveillance coverage		2.38(1.25)
	18	Security patrols		2.57(1.35)
	19	Traffic safety		3.77(0.85)

### 4.3 Exploratory factor analysis

Previous studies have indicated that using the same sample for both exploratory factor analysis (EFA) and confirmatory factor analysis (CFA) may lead to overfitting and compromise the reliability of results (81, 86). Therefore, this study used SPSS 22.0 to randomly split the full sample into two subsamples (A and B), with subsample A used for EFA and subsample B used for CFA. The resulting subsample A consisted of 226 cases, and subsample B consisted of 206 cases, both of which met the required criteria. The validity tests of subsample A showed a KMO value of 0.896 (>0.6), and Bartlett's test of sphericity was significant (*p* = 0.000 < 0.01), indicating that the data were suitable for exploratory factor analysis.

The principal component analysis was employed to extract characteristic variables from the 19 observed variables. A varimax rotation was applied to the rotation matrix. Based on the criterion of eigenvalues greater than 1, seven new evaluation factor groups and their compositions were initially extracted from the 19 observed variables (Table 4). However, as the principal components represented by the variables within Factor 6 and Factor 7 exhibited low explanatory power, making them unsuitable for forming new evaluative factor groups, the observed variables contained within these two factors were eliminated. The adjusted five-factor structure all passed validity analysis, with a cumulative contribution rate of 67.88%, encompassing over 60% of the information from the questionnaire items. Therefore, these five new factor groups can be considered to represent the majority of the information contained within the items. They were named according to the connotations of their respective observed variables as follows: Site Safety, Management Safety, Neighborhood Safety, Facility Safety, and Environmental Safety.

**Table 4:** Rotated component matrix of observed variables for children's outdoor activity space evaluation.

No.	Observed variable	Cumulative variance explained (%)							Cumulative variance explained
		1	2	3	4	5	6	7	
1	Site cleanliness	0.835	0.299	0.23	0.002	−0.003	−0.04	−0.012	Site safety 20.73%
2	Site openness	0.811	0.247	0.197	0.238	0.021	−0.051	0.007	
3	Greening & aesthetics	0.749	0.337	0.215	0.187	0.018	0.022	0.027	
4	Sunlight exposure	0.735	0.208	0.103	0.339	0.041	0.007	−0.06	
5	Site flatness	0.627	0.112	0.381	0.098	−0.14	0.058	0.039	
6	Strict access control	0.266	0.837	0.072	0.208	0.022	−0.092	0.006	Management safety 37.37%
7	Security patrols	0.214	0.831	0.103	0.245	0.073	0.033	−0.061	
8	Surveillance coverage	0.268	0.829	0.095	0.015	0	0.047	0.065	
9	Fear of stranger	0.304	0.674	0.449	0.112	−0.013	0.046	−0.015	
10	Availability of playmates	0.259	0.102	0.861	0.02	0.044	0.033	−0.017	Neighborhood security 51.22%
11	Neighborhood harmony	0.175	0.105	0.791	0.201	−0.064	0.064	0.037	
12	Community activity participation	0.247	0.181	0.74	0.211	−0.039	−0.111	−0.038	
13	Rest facilities	0.316	0.258	0.283	0.693	0.053	0.045	−0.024	Facility safety 60.00%
14	Facility maintenance	0.528	0.23	0.265	0.624	0.005	0.01	0.021	
15	Accessibility facilities	0.514	0.359	0.199	0.548	0.001	−0.024	−0.018	
16	Traffic safety	0.04	0.081	0.019	−0.156	0.889	0.011	−0.029	Environmental safety 67.88%
17	Public security conditions	−0.069	−0.024	−0.083	0.235	0.807	−0.009	0.213	
18	Spatial enclosure	−0.011	0.012	0.012	0.021	0.003	0.991	0.029	73.28%
19	Residential scale	0.004	0.009	−0.003	−0.017	0.14	0.03	0.979	78.67%

The reliability, as measured by Cronbach's α coefficient, was 0.900 (>0.7). The validity, indicated by the Kaiser–Meyer–Olkin (KMO) measure of sampling adequacy, was 0.896 (>0.7), with a significance level of p = 0.000 (< 0.01). The extraction method employed was Principal Component Analysis (PCA), and the rotation method utilized was Varimax with Kaiser Normalization.

### 4.4 First-order confirmatory factor analysis

**Figure 3:**
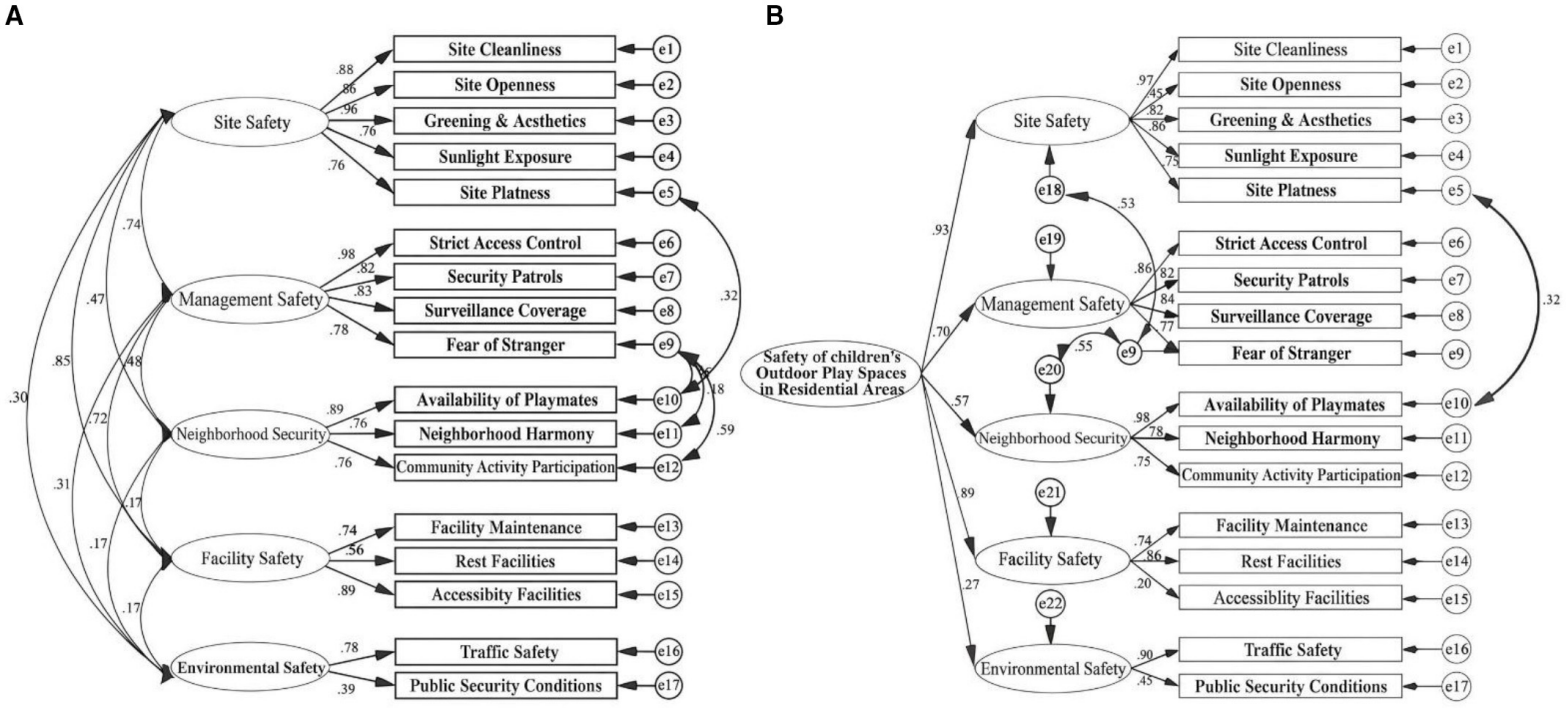
**(A)** Initial hypothesized model for assessing the safety of children's outdoor play spaces in residential areas. **(B)** Revised standardized parameter estimation path diagram for the safety assessment of children's outdoor play spaces in residential areas.

Based on the five new factor groups obtained from the aforementioned exploratory factor analysis, a preliminary safety evaluation model for children's outdoor activity spaces was constructed. The latent variables include Site Safety, Management Safety, Neighborhood Safety, Facility Safety, and Environmental Safety. Each latent variable corresponds to its respective observed variables. The arrows in the path diagram represent the interrelationships among the latent variables, and a residual term is established for each observed variable (Figure 3A). First-order confirmatory factor analysis was conducted using the maximum likelihood method in AMOS 21.0 software.

The reliability assessment, conducted through first-order confirmatory factor analysis using Average Variance Extracted (AVE) and Composite Reliability (CR) (Equations 1, 2), demonstrated that all observed variables exhibited CR values exceeding the 0.6 threshold and AVE values above the 0.5 criterion, meeting fundamental evaluation standards (85). All 19 observed variables showed statistical significance (Table 5). Regarding model fit indices, metrics including chi-square (χ^2^/df), CFI and NNFI indicated acceptable values. Although the RMR, GFI, and RMSEA approached acceptable thresholds, they required improvement. To enhance model fit, sequential modifications were implemented with reference to Modification Indices (MI), establishing correlations between residual terms of specific variables. The final modified model indicated that the χ^2^/df ratio falls within the acceptable range of 1–3, that the GFI and RMR indices are close to the recommended cut-off values, and that all other indices meet the required criteria, suggesting that the overall model fit is satisfactory (Table 6).


$$\begin{array}{l} AVE=\frac{\sum \lambda^{2}}{n},\text{λ}=\text{factor loadings},\text{n}=\text{the number of}\\ \text{ measurement indicators for this factor} \end{array}$$ (1)

$$\begin{array}{lll} CR & = & \frac{{\left(\sum \lambda \right)}^{2}}{{\left(\sum \lambda \right)}^{2}+\sum \delta },\text{λ and δ are standardized values} \end{array}$$ (2)

**Table 5:** First-order confirmatory factor analysis and reliability values of each factor.

Factor	Observed variable	Cronbach's α	AVE	Composite reliability (CR)	Unstandardized loading	Standardized loading	*Z*	S.E.	*p*
Site safety	Site cleanliness	0.899	0.714	0.926	1.000	0.875	-	-	-
	Site openness				0.979	0.856	16.672	0.059	0.000^***^
	Greening & aesthetics				0.987	0.875	17.410	0.057	0.000^***^
	Site flatness				0.985	0.860	16.802	0.059	0.000^***^
	Sunlight exposure				0.765	0.752	13.279	0.058	0.000^***^
Management safety	Strict access control	0.877	0.687	0.898	1.000	0.872	-	-	-
	Security patrols				0.927	0.817	14.583	0.064	0.000^***^
	Surveillance coverage				0.858	0.828	14.890	0.058	0.000^***^
	Fear of stranger				0.520	0.797	14.021	0.037	0.000^***^
Neighborhood safety	Availability of playmates	0.825	0.665	0.856	1.000	0.891	-	-	-
	Neighborhood harmony				0.902	0.770	12.210	0.074	0.000^***^
	Community activity participation				0.880	0.780	12.381	0.071	0.000^***^
Facility safety	Rest facilities	0.849	0.693	0.871	1.000	0.739	-	-	-
	Facility maintenance				1.190	0.890	12.687	0.094	0.000^***^
	Accessibility facilities				1.192	0.861	12.304	0.097	0.000^***^
Environmental safety	Traffic safety	0.640	0.460	0.626	1.000	0.756	-	-	-
	Public security conditions				0.801	0.579	3.148	0.254	0.002^**^

In general, an AVE above 0.5 or a CR above 0.7 indicates acceptable convergent validity; only one of these criteria needs to be met. ^***^, ^**^, and ^*^ denote significance at the 1%, 5%, and 10% levels, respectively.

**Table 6:** Model fit indices.

Common indices	*x^2^*	*df*	*p*	*χ^2^*/*df*	GFI	RMSEA	RMR	CFI	NFI	NNFI
Criteria	-	-	>0.05	< 3	>0.9	< 0.10	< 0.05	>0.9	>0.9	>0.9
First-order initial model	265.714	109	0	2.438	0.873	0.084	0.063	0.936	0.897	0.936
Assessment	-	-	-	Fair	Unmet	Unmet	Unmet	Fair	Unmet	Fair
First-order modified model	215.035	105	0	2.048	0.895	0.071	0.068	0.955	0.916	0.942
Assessment	-	-	-	Fair	Unmet	Fair	Unmet	Superior	Fair	Fair
Second-order model	204.039	111	0	1.838	0.900	0.062	0.064SRMR = 0.049	0.962	0.921	0.953
Assessment	-	-	-	Superior	Fair	Fair	Fair	Superior	Fair	Superior

### 4.5 Second-order confirmatory factor analysis

The correlation coefficients from the first-order confirmatory factor analysis indicated strong interrelationships among the first-order factors, suggesting the existence of a higher-order construct—namely, the overall safety of children's outdoor activity spaces in residential areas. Consequently, a second-order confirmatory factor analysis model was constructed. The maximum likelihood method was employed for parameter estimation, resulting in a standardized estimation model (Figure 3B). The model fit indices are summarized in Table 6. Given that RMR (root mean square residual) is sensitive to the scale of variables, the standardized RMR (SRMR) was additionally examined and found to be 0.048 (< 0.05), meeting the required threshold and indicating a good fit for the second-order model.

The standardized model demonstrated that all path coefficients between the overall safety evaluation and the five dimensional factors were statistically significant. The model structure revealed that site safety, management safety, neighborhood safety, facility safety, and environmental safety all had positive effects on the overall safety evaluation. Among the first-order factors, site safety exhibited the highest factor loading (0.93), indicating that this dimension has the strongest explanatory power and supporting role in evaluating the safety of outdoor activity spaces, and represents a core factor influencing users' perceived safety. In the safety evaluation model of outdoor activity spaces, the effects of site safety, facility safety, management safety, neighborhood safety, and environmental safety decrease in sequence. Notably, environmental safety showed a relatively low factor loading of only 0.27, suggesting that the overall residential environmental safety has a limited influence on the safety of internal outdoor activity spaces, with users paying greater attention to the safety conditions of activity sites themselves and their directly related factors. Overall, the observed variables demonstrate strong associations with the latent constructs, indicating that the sample data adequately support model construction. The model exhibits satisfactory fit, as well as good explanatory power and reliability.

From the perspective of interrelationships among variables, the model identifies three significant associations with correlation coefficients of 0.55, 0.53, and 0.32. The strongest relationship is between neighborhood safety and fear of strangers (0.55). Although fear of strangers is categorized under management safety in the model and has a standardized factor loading of 0.77, it is also strongly linked to perceived neighborhood safety, as neighborhood familiarity and social interaction shape users' perceptions of stranger-related risks. When neighborhood interaction and informal social monitoring are weak, users are more likely to perceive potential threats from strangers. A relatively strong association is also found between fear of strangers and site safety (0.53), suggesting that perceptions of stranger-related risks are influenced by spatial characteristics such as openness, visibility, and surveillability, which affect users' sense of control and overall perceived safety. In addition, a moderate correlation is observed between adequate sunlight and children having playmates (0.32), indicating that better sunlight conditions may encourage children to stay longer outdoors, thereby increasing peer interaction, spatial vitality, and perceived safety through enhanced natural surveillance.

### 4.6 Indicator weights

To determine the influence of each variable on the overall evaluation model, the weights of the indicators were calculated to assess the relative importance of the 17 first-order observed variables and 5 second-order latent variables on the safety evaluation of children's outdoor activity spaces in residential areas. Drawing on established methodologies for constructing evaluation systems using Structural Equation Modeling (SEM), this study employed factor analysis to calculate the weights. Factor analysis is an objective weighting method that mitigates the subjective bias inherent in subjective weighting approaches such as the Analytic Hierarchy Process (AHP).

The standardized path coefficients within the SEM represent the correlation between the indicators and their corresponding latent variables. The weights of the indicators were derived by normalizing these path coefficients. In this process, a~i~ represents the overall effect of the i-th latent variable on the safety evaluation (Equation 3). The weights of the observed variables were determined using the same method applied to the latent variables. This study ultimately established the weights for all latent and observed variables within the safety evaluation model for children's activity spaces in residential areas, as presented in Table 7.


$$\begin{array}{l} T_{i\;}=\frac{a_{i}}{\sum_{i=1}^{n}a} \end{array}$$ (3)

**Table 7:** Indicator weights of the safety assessment model for community outdoor play spaces.

Latent variable	Total effect	Level-1 weight	Observed variable	Factor loading	Level-2 weight	Rank
Site safety	0.93	0.27	Site cleanliness	0.87	0.21	1
			Site openness	0.85	0.20	5
			Greening & aesthetics	0.87	0.21	1
			Sunlight exposure	0.75	0.18	7
			Site flatness	0.86	0.20	3
Management safety	0.79	0.23	Strict access control	0.88	0.27	8
			Security patrols	0.82	0.25	11
			Surveillance coverage	0.84	0.25	9
			Fear of stranger	0.77	0.23	12
Neighborhood safety	0.57	0.17	Availability of playmates	0.88	0.37	13
			Neighborhood harmony	0.75	0.31	15
			Community activity participation	0.78	0.32	14
Facility safety	0.89	0.26	Facility maintenance	0.86	0.35	6
			Rest facilities	0.74	0.30	10
			Accessibility facilities	0.89	0.36	4
Environmental safety	0.27	0.08	Public security conditions	0.49	0.35	17
			Traffic safety	0.90	0.65	16

The weights of the primary indicators, in descending order, are as follows: Site Safety (0.93), Facility Safety (0.89), Management Safety (0.79), Neighborhood Safety (0.57), and Environmental Safety (0.27). Among these, site safety and facility safety carry the highest weights in the evaluation system, highlighting the critical importance of spatial planning, design, and facility arrangement in outdoor activity spaces for children's safety. In contrast, environmental safety has the lowest weight (0.27), indicating that the overall environmental conditions of residential communities have a relatively limited influence on parents' judgments regarding whether children can safely engage in outdoor activities. Compared with the broader residential environment, parents place greater emphasis on the internal spatial quality of the activity sites actually used by children and their directly related safety factors. This finding further suggests that the internal environment of activity spaces is a key dimension shaping perceived safety among both parents and children. In addition, management safety and neighborhood safety also exert strong influences on overall safety evaluation. Effective residential management can enhance spatial order and risk control capacity, while a positive neighborhood atmosphere and social interaction among residents contribute to higher levels of trust and informal social surveillance, thereby improving perceived safety in residential outdoor activity spaces to some extent.

## 5 Discussion

### 5.1 Safety perception framework and public health implications

This study constructed a safety evaluation framework for children's outdoor activity spaces based on five dimensions: site safety, management safety, neighborhood safety, facility safety, and environmental safety, and identified the relative importance of each dimension in overall safety perception. The results indicate that site safety and facility safety carry the greatest weights, suggesting that parents' and children's safety judgments are primarily derived from direct perceptions of the physical environment. Physical characteristics such as spatial layout, site boundaries, activity organization, and facility configuration constitute the core factors influencing safety cognition. Among these dimensions, site safety shows the highest weight, indicating that planning and design quality plays a fundamental role in shaping safety perception. Previous studies have demonstrated that attributes such as spatial visibility, boundary clarity, and traffic separation directly affect parents' assessments of children's activity risks (29, 59, 87). The present findings further suggest that safety in children's activity spaces is not only related to objective risk control, but also represents a subjective psychological perception formed through spatial experience. Particularly in high-density residential communities, the enclosure, accessibility, and spatial order of activity spaces significantly influence parents' willingness to allow children to engage in independent outdoor activities (2, 43, 88). Facility safety ranks second only to site safety, indicating that facility condition, age appropriateness, and the provision of barrier-free supporting facilities are also important determinants of safety perception. Because children interact directly with facilities, aging, damaged, or potentially hazardous facilities can significantly reduce evaluations of spatial safety. This finding suggests that the safety of children's activity spaces is more strongly associated with immediate perceptions of micro-scale facility environments than with broader macro-environmental quality.

The study also found that management safety and neighborhood safety significantly affect safety perception, although their effects are weaker than those of site safety and facility safety. Within the context of gated residential communities in China, community management and public order maintenance contribute to residents' perceptions of environmental stability (35, 72). Meanwhile, neighborhood familiarity, resident interaction, and social trust help alleviate parents' concerns regarding unfamiliar risks. These findings are consistent with the “social cohesion–safety perception” theory (65), which posits that positive neighborhood relationships and community trust enhance perceptions of environmental safety. In contrast, surrounding environment safety demonstrates the lowest weight, indicating that the overall environmental quality of residential communities has a relatively limited direct influence on children's safety perception. Compared with macro-level environmental image, parents and children pay greater attention to spatial and facility conditions directly related to actual activities, and safety perception is more strongly grounded in proximal and immediate spatial experiences.

From the perspective of public health research, the findings of this study further suggest that safety perception in children's outdoor activity spaces is not merely an issue of residential environmental evaluation, but is also closely associated with the formation of children's health behaviors and the development of health-supportive environments. Public health studies generally recognize outdoor activity as a critical behavioral foundation for promoting children's physical activity levels, psychological restoration, social interaction, and cognitive development (89, 90), while perceived safety constitutes a key environmental prerequisite for sustained participation in outdoor activities. Compared with objective risks themselves, parents' subjective perceptions of the safety of residential activity environments more directly influence the duration, frequency, and spatial autonomy of children's outdoor activities (23, 91). Therefore, safety perception can be understood as an important psychological–environmental mediating mechanism in the formation of children's health behaviors. The finding that site safety and facility safety dominate the overall safety perception framework further supports the concept of “micro-scale environmental priority” in built environment and public health research (36, 38), which suggests that children's health behaviors are more strongly dependent on environmental qualities that can be directly perceived and immediately experienced. Moreover, the conclusion that management safety and neighborhood safety also significantly influence safety perception is consistent with the theoretical logic of the Social Ecological Model in public health, which posits that children's health behaviors result from the interaction of individual characteristics, physical environments, and social environments (25, 92). Positive neighborhood interaction and social cohesion not only enhance safety perception, but also strengthen children's sense of community belonging and opportunities for social interaction, thereby exerting potentially positive effects on children's social development and psychological wellbeing. Furthermore, compared with traditional health evaluation paradigms based primarily on objective environmental exposure, this study emphasizes the explanatory value of subjective perception in the formation of health behaviors.

### 5.2 Practical applications

The weights assigned to each indicator in the safety evaluation system provide a empirical foundation for proposing targeted improvement strategies.

#### 5.2.1 Creating comfortable and well-maintained spatial environments

In terms of site safety, factors such as aesthetic greenery, cleanliness, spatial openness, surface evenness, and adequate sunlight exhibit relatively balanced influences on safety evaluations. Field observations reveal that continuous, open spaces not only accommodate children's innate desire for running and chasing activities but also provide safe environments for cycling and ball games, thereby promoting moderate-to-vigorous outdoor physical activities. Sufficient sunlight and visually pleasing green landscapes help create natural and comfortable activity environments, enhancing both parents' and children's sense of safety. Such spaces support leisure, learning, social interaction, psychological development, and cultural expression for children of all ages, ultimately contributing to their healthy development.

#### 5.2.2 Providing accessible and comprehensive recreational facilities

Regarding facility safety, the factors influencing users' perceived safety in descending order of importance are: accessibility facilities, facility maintenance, and rest facilities. Interviews revealed that since outdoor activities for infants and young children often involve equipment such as strollers and tricycles, the presence of accessibility facilities in activity areas can significantly improve both accessibility and safety. The study also found that the availability of rest facilities and the functionality of play equipment directly affect users' perceived safety, which in turn influences their frequency of use and duration of activities. Therefore, children's outdoor activity spaces in residential areas should be equipped with an appropriate number of rest facilities, and play equipment should be regularly maintained to reduce the risk of accidents, ensure children's safety, and promote their outdoor activities.

#### 5.2.3 Establishing a secure and well-organized community management system

In terms of management safety, factors such as access control strictness, stranger fear, security patrols, and surveillance equipment significantly influence parents' safety evaluations. Enhancing the coverage of surveillance equipment around outdoor activity spaces, increasing mobile security patrols, and strengthening security supervision can effectively improve monitoring of children's outdoor activities. These measures help elevate residents' psychological sense of security, reduce stranger-related anxiety, and promote a safer environment for children's independent activities.

#### 5.2.4 Fostering a harmonious community atmosphere through co-governance and shared benefits

Regarding neighborhood safety, the factors influencing users' perceived safety in descending order of importance are: availability of playmates for children, participation in community activities, and neighborhood harmony. Within the neighborhood environment, children serve as the most cohesive element. By providing facilities and spaces conducive to group activities for children and organizing diverse events, a child-centered communication network can be fostered. This approach, which leverages “children motivating families, and families transforming communities,” helps enhance residents' sense of belonging and neighborhood integration, thereby improving the safety of children's outdoor activity spaces.

### 5.3 Limitations

This study developed a safety evaluation system for children's outdoor activity spaces in residential communities that are widely prevalent in China, and explored the major associated variables and factors influencing safety perception. However, several limitations still exist and should be further addressed and refined in future research. First, due to constraints in sample size and geographic coverage, the findings may be subject to sampling bias. The data were collected from residential communities in the central urban area of Nanjing; given regional differences in socioeconomic conditions and cultural contexts, the generalizability of the results should be further tested in other cities and similar residential settings. Future studies could expand the sample and conduct cross-regional comparisons to improve external validity. Second, the study relies mainly on subjective perceptions from parents and children. Although parents were encouraged to consult with children when completing the questionnaire, some responses may still reflect parental perceptions only, which may introduce bias. Future research should incorporate objective data such as behavioral observations and GPS tracking to enable multi-source validation and improve robustness. Finally, based on cross-sectional data, this study examines associations between residential environments and perceived safety, but does not fully explore causal mechanisms linking environmental factors, perceived safety, and children's outdoor behaviors. Future research should further investigate these pathways in residential community contexts to strengthen theoretical and practical implications.

## 6 Conclusion

This study systematically organized and summarized spatial safety indicators within residential communities through field investigation and questionnaire surveys, and employed Structural Equation Modeling (SEM) to construct an evaluation system of children's outdoor activity space safety from five dimensions: site safety, management safety, neighborhood safety, facility safety, and environmental safety. The study reveals multidimensional relationships between residential environmental factors and perceived safety. In particular, internal site-related factors play a key role in shaping parents' and children's perceived safety in outdoor activities. Moreover, residential management and neighborhood atmosphere contribute to the formation of residents' trust in the residential environment, thereby influencing parents' judgments of children's safety in outdoor activities within residential communities. The findings offer a comprehensive framework for assessing the safety levels of these spaces, reflecting the psychological perceptions and subjective safety feelings of parents and children. The proposed evaluation system helps identify key issues related to the safety of existing activity spaces and provides a scientific basis for future improvement strategies and further research on spatial safety in residential areas.

## Data Availability

The raw data supporting the conclusions of this article will be made available by the authors, without undue reservation.
