# Supplementary material for: Research on the safety evaluation of children's activity space in urban residential areas
Source: Front Public Health. 2026 Jun 17;14:1832723. doi: 10.3389/fpubh.2026.1832723 (PMC13318570; doi:10.3389/fpubh.2026.1832723)
Supplement: Supplementary file 1 [file Supplementary_file_1.docx]

**Survey Questionnaire on Safety Perception of Children's Outdoor Activity Spaces in Residential Areas**

(Residential Area Name: ____________________)

**Dear Parent/Guardian,**

Welcome to this survey. We sincerely invite you and your child to participate. Please answer based on your child’s actual usage and feelings. For children aged 10 and above, self-completion is encouraged. Your responses are crucial for our research on the safety perception of residential children's outdoor activity spaces. This study is conducted for academic purposes only and does not involve any privacy disclosure. Thank you for your cooperation and support!

**Part I: Basic Information**

**Q1. Your relationship with the child?**

A. Parent

B. Grandparent

C. Other Guardian

D. Babysitter

E. Other: _______

**Q2. Your Gender?**

A. Male

B. Female

**Q3. Child's Age Group?**

A. Under 3 years old

B. 3-5 years old

C. 6-8 years old

D. 9-12 years old

E. Over 12 years old

**Q4. Child's Gender?**

A. Male

B. Female

###### Part II: Characteristics of Children's Outdoor Activities

Q5. When does your child usually engage in outdoor activities? (Multiple choices)

A. Morning (before 12:00)

B. Afternoon (12:00–18:00)

C. Evening (after 18:00)

**Q6. How many days per week does your child engage in outdoor activities on average?**

A. Almost never

B. 1–2 times

C. 3–4 times

D. Daily

**Q7. How long does your child typically spend on each outdoor activity session?**

A. Less than 15 minutes

B. 15–30 minutes

C. 30–60 minutes

D. 1–2 hours

E. More than 2 hours

**Q8. What types of activities does your child usually engage in during outdoor play? (Multiple choices)**

A. Riding bicycles/tricycles

B. Ball games (basketball, football, etc.)

C. Sand play

D. Running games / Hide-and-seek

E. Jump rope / Shuttlecock kicking

F. Using adult fitness equipment

G. Socializing with friends

H. Interacting with landscape elements (plants, water features, etc.)

I. Other: _______

**Q9. Who does your child usually play with?**

A. Alone

B. Accompanied by parents

C. With peers/playmates

D. With unfamiliar children

E. With pets

**Part III: Safety Perception Evaluation**

Instructions:​ Please evaluate the safety perception of the following environmental elements using a 5-point Likert scale. Circle or tick the number that best represents your feeling.

(-2 = Very Unsafe, -1 = Unsafe, 0 = Neutral, 1 = Safe, 2 = Very Safe)

| **Category** | **Observed Variables** | **Safety Perception** | | | | | |
| --- | --- | --- | --- | --- | --- | --- | --- |
| **Activity Facility** | **Accessible Facilities** | | 2 | -1 | 0 | 1 | 2 |
|  | **Facility Maintenance** | | -2 | -1 | 0 | 1 | 2 |
|  | **Rest Facilities** | | -2 | -1 | 0 | 1 | 2 |
| **Activity Sites** | **Greening & Aesthetics** | | -2 | -1 | 0 | 1 | 2 |
|  | **Site Cleanliness** | | -2 | -1 | 0 | 1 | 2 |
|  | **Site Flatness** | | -2 | -1 | 0 | 1 | 2 |
|  | **Site Openness** | | -2 | -1 | 0 | 1 | 2 |
|  | **Sunlight Exposure** | | -2 | -1 | 0 | 1 | 2 |
| **Surrounding Environment** | **Spatial Enclosure** | | -2 | -1 | 0 | 1 | 2 |
|  | **Residential Scale** | | -2 | -1 | 0 | 1 | 2 |
| **Neighborhood Interaction** | **Community Activity Participation** | | -2 | -1 | 0 | 1 | 2 |
|  | **Availability of Playmates** | | -2 | -1 | 0 | 1 | 2 |
|  | **Neighborhood Harmony** | | -2 | 1 | 0 | 1 | 2 |
|  | **fear of strangers** | | -2 | -1 | 0 | 1 | 2 |
| **Security Management** | **public security conditions** | | -2 | -1 | 0 | 1 | 2 |
|  | **strict access control** | | -2 | -1 | 0 | 1 | 2 |
|  | **surveillance coverage** | | -2 | -1 | 0 | 1 | 2 |
|  | **security patrols** | | -2 | -1 | 0 | 1 | 2 |
|  | **Traffic Safety** | | 2 | -1 | 0 | 1 | 2 |
| **Overall Safety Perception of Children's Outdoor Activity Spaces** | | -2 | | -1 | 0 | 1 | 2 |

Finally, thank you again for your cooperation. We wish your child healthy growth and your family happiness.

**住区儿童户外活动空间安全性调研问卷**

（小区名称： ）

亲爱的家长，您好！我们真诚地邀请您和孩子参与此次调研，请您结合儿童的使用感受来填写，10岁以上儿童鼓励自己填写。您的真实想法和回答对我们的研究十分重要，本次研究内容仅用于关于住区儿童户外活动空间安全感知的科研论文，不涉及您隐私。感谢您的配合与支持！

**第一部分：基本信息调查**

1. **您是孩子的**_____？

A. 爸爸妈妈 B. 爷爷奶奶/外公外婆 C. 其他监护人 D. 保姆 E. 其他：

1. **您的性别？_**____ A. 男 B. 女
2. **您的孩子年龄？**_____

A. 3岁以下 B. 3-5岁 C. 6-8岁 D. 9-12岁 E. 12岁以上

Q4.**您孩子性别？**_____ A. 男 B. 女

**第二部分：儿童户外活动情况调查**

1. **您的孩子一般在哪个时间段活动？（多选）**_____

A. 早晨 B. 上午 C. 中午 D. 下午 E. 晚上(18:00后)

1. **您的孩子平均一周会有几天进行户外活动？_**____

A. 几乎没有 B. 1-2 C. 3-4 D. 5-6 E. 每天

1. **您的孩子每次活动的时间是多少？**_____

A. 15分钟内 B. 15-30分钟 C. 30分钟-1小时 D. 1小时以上

1. **您的孩子玩耍时会进行哪些活动？（多选）**_____
2. 骑车 B. 轮滑 C. 球类活动 D. 跳舞 E. 跳绳、踢毽子 F. 挖沙坑 G. 追逐打闹、捉迷藏 H. 儿童活动器材 I. 成年人健身器材 J. 与好友聊天 K. 与景观互动 L. 其他：
3. **您的孩子通常是和谁一起玩耍？**_____

A. 独自一人 B. 家长陪同 C. 小伙伴 D. 陌生儿童 E. 宠物

**第三部分：请您对户外活动场地构成要素进行安全感受评价**

请您对以下户外活动场地构成元素进行**安全感受评价**。（直接圈出或打勾即可）。感觉相关要素对场所的影响很不安全=-2，不太安全=-1，无感受=0，比较安全=1，非常安全=2。

| **评价方面** | **评价内容** | **安全感受** | | | | |
| --- | --- | --- | --- | --- | --- | --- |
| **A.活动设施** | A1无障碍设施 | -2 | -1 | 0 | 1 | 2 |
|  | A2活动设施及维护 | -2 | -1 | 0 | 1 | 2 |
|  | A3休憩设施 | -2 | -1 | 0 | 1 | 2 |
| **B.活动场地** | B1绿化美观 | -2 | -1 | 0 | 1 | 2 |
|  | B2场地卫生 | -2 | -1 | 0 | 1 | 2 |
|  | B3场地平整 | -2 | -1 | 0 | 1 | 2 |
|  | B4场地开阔 | -2 | -1 | 0 | 1 | 2 |
|  | B5日照充足 | -2 | -1 | 0 | 1 | 2 |
| **C.周边环境** | C1 空间围合 | -2 | -1 | 0 | 1 | 2 |
|  | C2 住区规模 | -2 | -1 | 0 | 1 | 2 |
| **D.邻里互动** | D1社区活动参与 | -2 | -1 | 0 | 1 | 2 |
|  | D2儿童有无玩耍伙伴 | -2 | -1 | 0 | 1 | 2 |
|  | D3邻里和睦 | -2 | -1 | 0 | 1 | 2 |
|  | D4陌生人恐惧 | -2 | -1 | 0 | 1 | 2 |
| **E.治安管理** | E1治安情况 | -2 | -1 | 0 | 1 | 2 |
|  | E2门禁严格 | -2 | -1 | 0 | 1 | 2 |
|  | E3 监控设备 | -2 | -1 | 0 | 1 | 2 |
|  | E4 保安巡逻 | -2 | -1 | 0 | 1 | 2 |
|  | E5交通安全 | -2 | -1 | 0 | 1 | 2 |
| 对在本社区活动安全感受评价 | | -2 | -1 | 0 | 1 | 2 |

最后，再次感谢您的配合，祝您的孩子健康成长。家庭幸福美满！
